# Intracellular mechanisms of solar water disinfection

**DOI:** 10.1038/srep38145

**Published:** 2016-12-02

**Authors:** María Castro-Alférez, María Inmaculada Polo-López, Pilar Fernández-Ibáñez

**Affiliations:** 1 Plataforma Solar de Almería – CIEMAT, P.O. Box 22, 04200 Tabernas (Almería), Spain; 2 CIESOL, Joint Centre of the University of Almería-CIEMAT, 04120 Almería, Spain

## Abstract

Solar water disinfection (SODIS) is a zero-cost intervention measure to disinfect drinking water in areas of poor access to improved water sources, used by more than 6 million people in the world. The bactericidal action of solar radiation in water has been widely proven, nevertheless the causes for this remain still unclear. Scientific literature points out that generation of reactive oxygen species (ROS) inside microorganisms promoted by solar light absorption is the main reason. For the first time, this work reports on the experimental measurement of accumulated intracellular ROS in *E. coli* during solar irradiation. For this experimental achievement, a modified protocol based on the fluorescent probe dichlorodihydrofluorescein diacetate (DCFH-DA), widely used for oxidative stress in eukaryotic cells, has been tested and validated for *E. coli*. Our results demonstrate that ROS and their accumulated oxidative damages at intracellular level are key in solar water disinfection.

Solar water disinfection (SODIS) is one of the most practical and low-cost techniques to reduce the load of pathogenic microorganisms in water at households in low-income areas. It consists on the exposure of polluted water in 1.5–2L-PET (polyethylene terephthalate) transparent containers to full sunshine for at least 6 h^1^. SODIS was known and used nearly 2000 years ago by Indian communities to purify drinking water. The bactericidal effect of sunlight was experimentally studied for the first time by Downes & Blunt in 1877^2^. The first successful application was demonstrated by Acra *et al*. in 1980^3^. Since then, SODIS accounts for more than 300 research articles. The mechanism of action of this photo-induced process has been intensively investigated since nineties till now. Most of research was focused on the influence of key parameters like water temperature^4^,^5^, solar energy dose^6^, turbidity of polluted water^7^, dissolved oxygen^8^, nature of the microorganism^9^, and other studies on the practical application of this technique and field trials in several locations^10^,^11^,^12^,^13^. Other contributions report research and technical developments in solar reactors to use SODIS for scaled-up solutions^1^,^14^,^15^.

Bactericidal effect of sunlight is commonly attributed to the synergistic effect of solar UV photons and mild-thermal heating produced during exposure. It is accepted that UV-B causes damages in DNA by direct absorption of light by endogenous chromophores^1^. Accumulated damages induced by Reactive Oxygen Species (ROS) generated by solar UV-A photons inside bacterial cells are also considered responsible for the damage produced by solar radiation^16^. ROS comprehends some free radicals, such as superoxide (O_2_^•−^), hydroperoxyl (HO_2_^•^), hydroxyl (HO^•^), peroxyl (ROO^•^), alkoxyl (RO^•^), and also non-radicals species, such as hydrogen peroxide (H_2_O_2_), singlet oxygen (^1^O_2_), and hypochlorous acid (HOCl). ROS have been proven to induce lipids peroxidation, proteins oxidation, DNA damages, by formation of pyrimidine dimers, or generation of single strands breaks^17^. Although, it is accepted that ROS play a very important role in solar disinfection, to our knowledge there is not any study focused in determining experimental evidence of intracellular ROS increase in bacteria by solar exposure.

Nevertheless, other research fields have done big achievements on ROS and RNS (radical nitrogen species) detection in cells tissues for medical applications. ROS and RNS generated endogenously or in response to environmental stress have long been implicated in tissue injury in the context of a variety of disease states^18^. Cancer therapy, photodynamic therapy and antimicrobial infections have given a number of contributions on oxidative stress determination in eukaryotic cells. For this purpose, specific molecules (probes) with high selectivity to ROS and generating fluorescent products have been used^19^,^20^,^21^.

There is a wide range of fluorescent probes; selecting the most suitable for a specific ROS detection and also for a type of cell, cellular oxidative stress method or a ROS target is not an easy task. Very few organic molecules react stoichiometrically with radicals and oxidant species to form detectable intracellular fluorescent products. Main fluorescence probes are classified as a function of the species to which they are more reactive. For detection of superoxide radical, dihydroethidium, 1,3-diphenylisobenzofuran, and 2-(2-pyridil)-benzothiazoline are used. Singlet oxygen is detected using 9,10-dimethylanthracene and 9-[2-(3-carboxy-9,10-diphenyl)anthryl]−6-hydroxy-3H-xanthen-3-ones, among others. There are fluorescence probes for hydroxyl radical detection like 4-(9-anthroyloxy)−2,2,6,6-tetramethylpiperidine-1-oxyl, 1,3-cyclohexanedione, sodium terephthalate, coumarin, fluorescein, etc. Peroxyl radical can be detected with cis-parinaric acid, lipophilic fluorescein derivatives, 2,7-dichlorodihydrofluorescein diacetate, etc. For detection of hydrogen peroxide, 2,7-dichlorodihydrofluorescein, scopoletin, and amplex red are selected^22^.

The most commonly used probe as indicator of intracellular oxidative stress is 2,7-dichlorodihydrofluorescein diacetate (DCFH-DA)^22^. It is a good indicator of hydroxyl radicals (HO^•^), hydrogen peroxide (H_2_O_2_) and peroxyl radicals (ROO^•^)^23^. It can diffuse through the cellular membrane and it is naturally hydrolyzed by endogenous cell esterases removing diacetate groups and forming 2,7-dichlorodihydrofluorescein (DCFH). Then, non-fluorescent DCFH originates 2,7-dichlorofluorescein (DCF) by indirect reactions with iron, heme proteins, GS^•^, CO_3_^•−^, NO_2_^•^, HO^•^, and H_2_O_2_ (Fig. 1). DCF is a fluorescent compound that can be detected by absorbance spectroscopy at 500 nm (ε = 59500 M^−1^ cm^−1^) and fluorescence spectroscopy at λ_emission_ = 522 nm and λ_excitation_ = 498 nm^19^.

Intracellular redox chemistry of DCFH-DA obeys to a very complex group of reactions that generate intermediates like DCF^•^, DCFH^•^, DCF^•−^, (DCF)^*^, which also react with one-electron-oxidizing species. Other compounds and radicals involved in these reactions include O_2_, O_2_^•−^, NADH, redox-active metals like copper and iron, cytochrome c, enzymes including peroxidase, etc. The presence of these substances interferes the formation of DCF making this probe to be considered better as a marker of the cellular oxidative stress than as indicator of the formation of H_2_O_2_ or other ROS and RNS^22^,^25^. Even DCFH is used as an oxidative burst indicator in macrophages and neutrophils^26^.

The main goal of this work is to find out experimental evidence of ROS generation in *E. coli* bacteria in water exposed to solar radiation. Firstly, a protocol to detect intracellular generated ROS in *E. coli* cells using DCFH-DA as ROS probe has been developed. Several aspects related to the use of this probe such as its chemical stability under solar radiation, hydrolysis, suitable concentration and reaction time to favor optimal response among probe and bacteria were investigated in this work. As a result, a new protocol for intracellular ROS detection in solar irradiated *E. coli* in water was proposed and validated. This protocol was eventually used to evaluate and detect the photo-induced generation of internal ROS in *E. coli* cells exposed to solar radiation.

## Methods

### Chemicals

The probe 2,7-dichlorodihydrofluorescein diacetate (DCFH-DA, Sigma Aldrich) was used as received and dissolved in dimethyl sulfoxide (DMSO, Sigma Aldrich). Sodium hydroxide (NaOH, J. T. Burker) and phosphate-buffered saline (PBS, Oxoid) were used for probe pre-treatment. Hydrogen peroxide (H_2_O_2_, 35% w/v, Sigma Aldrich) was used as received for positive control experiments. Luria-Bertani nutrient medium (LB Broth, Panreac) and Luria-Bertani agar (LB agar, Panreac) were used for bacterial growth and quantification.

### Bacterial strain enumeration and quantification

*E. coli* K12 strain was obtained from the Spanish Culture Collection (CECT) and used for experiments in distilled water spiked with seeded bacteria. Fresh liquid cultures were prepared in LB Broth and incubated at 37 °C with rotary shaking for 20 h to get stationary phase. Bacterial concentration in this growth stage was 10^9^ CFU mL^−1^. Then, bacterial suspensions were harvested by centrifugation at 900 × *g* for 10 min and bacterial pellet was re-suspended in PBS solution and diluted to the initial concentration of the experiments directly in the reactor. The samples taken during the experiments were enumerated using the standard plate counting method using LB agar, through 10-fold serial dilutions in PBS with a detection limit of 2 CFU mL^−1^. Colonies were counted after incubation for 24 h at 37 °C.

### Fluorescence detection

In this work, different types of equipment were used for DCF fluorescent signal detection: (i) Fluorescence microscope Leica DM 2500 with excitation filters of 480/40 and emission filters of 585/40 coupled to a DFC365 FX camera. (ii) Flow cytometer (FACSCANTO II) using a blue light that excites the samples (488 nm) and detection filters LongPass LP502 and Bandpass 530/30. The equipment gives the fluorescent signal of ca. 50 000 bacteria cells in terms of FITC-A signal. FITC-A (Fluorescein isothiocyanate) is a very well-known fluorescent probe used in cytometric assays and whose signal is used as arbitrary units as reference to other probes as DCFH that emits in the same wavelength. (iii) Fluorimeter (Spectrafluor Plus), excitation/emission: 497/520 nm that provides the fluorescent signal in terms of CPS (counts per second). (iv) Spectrophotometer (Unicam-II) used to perform spectral scanning in the range of wavelength of 300–800 nm with a quartz cuvette of 1 cm of path length.

### Solar irradiation tests

Two types of solar irradiation tests were done: (i) DCFH-DA exposed to simulated sunlight, and (ii) solar water disinfection (with *E. coli*) under natural sunlight.

Previous to natural solar disinfection experiments, studies of probe photo-degradation were done using a simulated solar radiation equipment (SUNTEST XLS+, Atlas). The artificial sunlight was provided by a xenon lamp that has incorporated a daylight filter with the aim to simulate solar noon spectrum. The irradiance set point was set to 30 W m^−2^ in the UV-A range, which corresponds to 410 W m^−2^ in the range of 300–800 nm. The total suspension volume (20 mL) was exposed to simulated solar radiation in front of lamp direction in an open vessel. Water reactor was spiked with 50 μM of DCFH-DA before the light exposure. Samples were taken during the experiment to measure UV/vis absorption spectra.

Solar water disinfection experiments for the study of intracellular ROS formation during *E. coli* inactivation were done under natural sunlight in clear sky conditions in April 2016 at the solar facilities of CIEMAT (PSA), South of Spain. Temperature and solar irradiance were monitored during the solar exposure by a temperature sensor and an UV-A detector for outdoor operation (Solar Light CO., Inc. Laser Technology) that provides measurements of UV-A irradiance in the range of 320–400 nm in terms of W m^−2^. The solar disinfection experiments were done in 200 mL borosilicate vessels with a glass cover to avoid contamination and permit UV-A income to water samples. Initial concentration of bacteria was 10^6^ CFU mL^−1^ in all cases. The experiments were performed within autoclaved distilled water with 0.9% w/v of NaCl to avoid bacterial osmotic stress. Samples were taken during the experiments for *E. coli* enumeration and intracellular ROS detection. Four replicated tests were done of solar experiments. Average of results (mean value) and standard deviation (error) are reported in results section.

## Results

### General concept of proposed protocol for intracellular ROS detection

The procedure used in this work to detect intracellular ROS by fluorescent probes is based on the general mechanism of recognized ROS-probes^23^, and more specifically of DCFH-DA. In the present study three novel achievements will be developed and demonstrated to permit detecting significant intracellular ROS levels in solar irradiated bacteria in water. These are: (i) lab-hydrolysis of DCFH-DA prior to bacterial incubation, instead of natural hydrolysis by esterases in bacteria cells, which will guarantee perfect and faster interaction between DCFH and intracellular ROS; (ii) determination of optimal DCFH-DA concentration for a significant and reliable measurement of fluorescent signal for *E. coli* in water, and (iii) optimization of incubation time for the reaction of DCFH and intracellular *E. coli*-ROS to obtain time-stable response of fluorescent signal (flow cytometry and fluorescent microcopy).

### Photo-chemical stability of DCFH-DA

Previous DCFH-DA protocols proposed an incubation period with cells^23^, then they are exposed to the oxidative stress under assessment. In the case of bacteria exposed to solar radiation, the oxidative stress is produced by continuous income of solar UV photons over bacteria. Initially, exposure of DCFH-DA to solar radiation was considered, and therefore its photo-chemical stability was tested.

**Figure 2:**
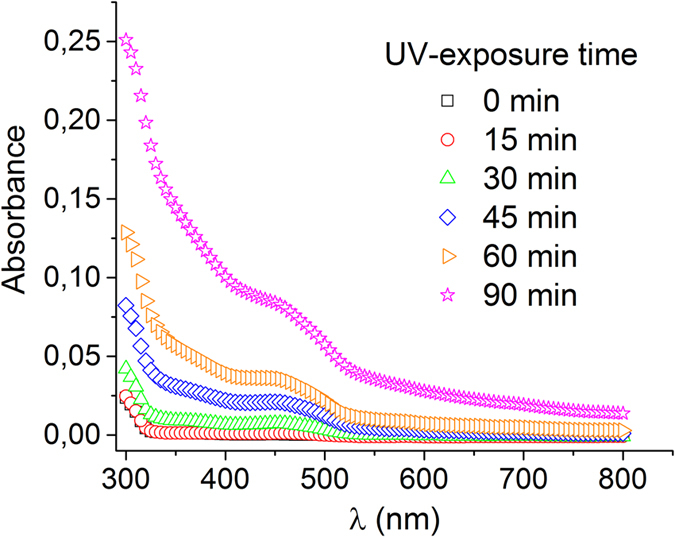
DCFH-DA photo-degradation. Absorbance spectrum of a 50 μM DCFH-DA solution at 0, 15, 30, 45, 60 and 90 min of solar radiation (30 W m^−2^ UV-A).

Absorbance spectrum of irradiated DCFH-DA (without bacteria) was measured at different times of solar exposure, from 0 to 90 min (Fig. 2). The absorbance increase in the range of 400–500 nm, suggested the formation of photo-transformation compounds. Similar observation was published by Chignell and Sik^27^, they measured an increase of DCF fluorescence signal in cell-free samples containing DCF, DCFH and horseradish peroxidase when irradiated by UV-A. This can be attributed to the photo-reduction of DCF, involving (DCF)^*^ and DFC^•−^ generation^1^,^3^. The formation of these photo-products could also alter the ROS levels due to the high reactivity of DFC^•−^ with oxygen, generating O_2_^•−^ and consequently H_2_O_2_. Hence, given the photo-transformation of DCFH-DA under sunlight, the addition of DCFH-DA to *E. coli* should be done only after solar exposure of bacteria in water. Although some researchers used the probe prior to cellular oxidative stress^28^,^29^, alternatively the probe might be used after if ROS generation is an UV-photo induced process, as reported for cyanobacterium *Anabaena* sp. under UV-B^30^,^31^.

### Hydrolysis of DCFH-DA

When bacterial cells are loaded with DCFH-DA, diacetate groups are hydrolyzed by endogenous esterase to generate DCFH. However, under irradiation, it is expected that many intracellular molecules are modified or even inactivated^1^,^32^,^33^. It is also assumed that esterase may be altered by solar radiation, and therefore it may be working with lower efficiency than in unaltered cells. Therefore, solar irradiated cells could be affected at esterase level, so that proper ROS detection by DCFH-DA is not optimal. To avoid this, an alternative chemical hydrolysis procedure is proposed to load the cells with DCFH instead of DCFH-DA. The advantage of pre-hydrolysis is not only to avoid potential esterases degradation, but also to permit ROS reaction with hydrolyzed DCFH-DA immediately after added to bacteria, avoiding longer periods of intracellular reactions.

Chemical hydrolysis of DCFH-DA is induced to remove diacetate groups from the molecule. Hydrolysis of DCFH-DA was done following the protocol described elsewhere^34^. Briefly, 0.01 N of NaOH was added to 50 μM of DCFH-DA and incubated 30 minutes in dark. Then, the probe was neutralized with PBS 0.1 M to obtain the hydrolyzed form, DCFH.

### DCFH-H_2_O_2_ reaction: concentration dependence

**Figure 3:**
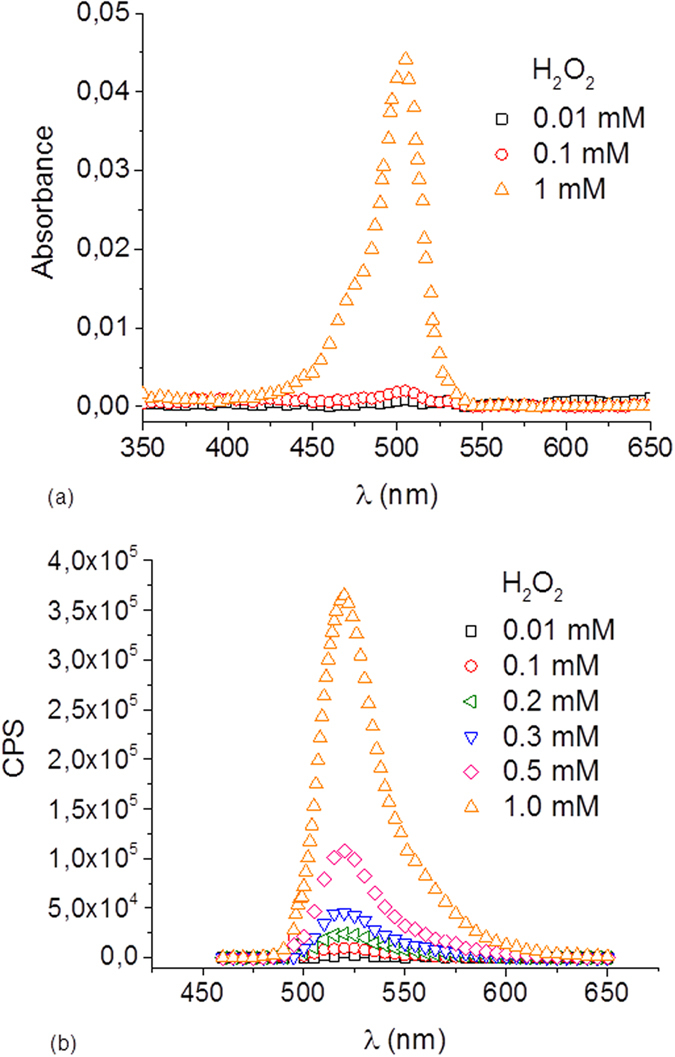
DCF concentration is dependent on H_2_O_2_ concentration. (**a**) Absorbance spectrum and (**b**) fluorescence emission signal (excitation: 497 nm) of the formed DCF product from the reaction between DCFH (10 μM) and several H_2_O_2_ concentrations.

Reaction of DCFH with ROS to produce DCF was measured by absorbance and fluorescence signals. In this case, H_2_O_2_ was used as a positive control of ROS. H_2_O_2_ was chosen due to its capability to react with DCFH and to penetrate easily into bacteria cells^35^. Furthermore, H_2_O_2_ is commonly accepted as one of the ROS that are generated inside bacteria under light stress^36^. Reaction between 10 μM DCFH and H_2_O_2_ at different concentrations (0.01 to 1 mM) was done in the absence of bacteria in dark. These concentrations were selected to ensure good measurement of absorbance and fluorescent signals in the spectrophotometer and fluorimeter respectively, and to check if the hydrolyzed probe was reactive to H_2_O_2_. Further experimental work will optimize concentrations of the probe to measure ROS at intracellular levels in *E. coli.* Figure 3 shows that DCF formation is H_2_O_2_-concentration dependent; the higher H_2_O_2_ concentration the higher DCF concentration was observed for both, absorbance and fluorescence peak values. This confirms that hydrolyzed DCFH by NaOH can be used as a good indicator of H_2_O_2_.

**Figure 4:**
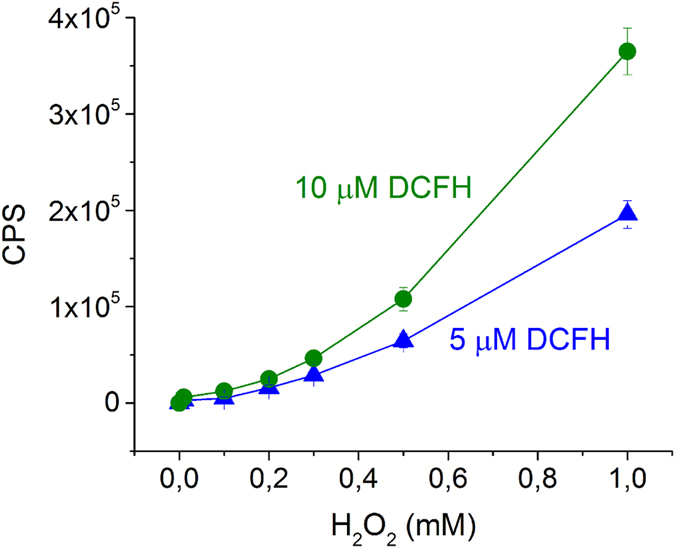
Effect of DCFH concentration in fluorescence measurements. Maximum fluorescence emission signal (excitation/emission: 497/520 nm) of DCF product formed from the reaction between 5 and 10 μM (chemically hydrolyzed) DCFH with H_2_O_2_ (0.01, 0.1, 0.2, 0.3, 0.5, and 1 mM).

In order to determine the saturation concentration of DCFH for a certain amount of H_2_O_2_, and to avoid overload of DCFH which may give undesired fluorescence background signal, several reactions of DCFH and H_2_O_2_, at variable concentrations of both, were carried out in absence of bacteria and measured with the fluorimeter. Concentrations of DCFH were 1, 5, 10, 15, and 20 μM with different concentrations of H_2_O_2_, 0.01, 0.1, 0.2, 0.3, 0.5 and 1 mM. At view of first results, 1, 15 and 20 μM-DCFH were discarded, as the signal was too low for 1 μM, while it was unstable at 15 and 20 μM, probably caused by the effect of ambient light and oxygen. Two replicates of reactions at 5 and 10 μM of DCFH with all concentrations of H_2_O_2_ were done, and resulted highly reproducible (confidence level > 99%). Average values are shown in Fig. 4.

At low H_2_O_2_ concentrations, the fluorescence signal for both, 5 and 10 μM of DCFH, led to very similar values, while at higher H_2_O_2_ concentrations (>0.3 mM) 10 μM of DCFH showed higher values than for 5 μM, being 10 μM more precise to detect differences in fluorescence measurements. Due to the stability of the measurements at all H_2_O_2_ concentrations tested, both DCFH concentrations (5 and 10 μM) could be a good option to determine the internal ROS in *E. coli*. In this work, 10 μM of DCFH was selected for further experiments to detect fluorescence signal caused by intracellular generated ROS in solar exposed *E. coli*. Although *E. coli* intracellular ROS values are expected to be very low as compared to this previous calibration; for instance 0.01 μM H_2_O_2_ intracellular concentration was reported^37^, it is more accurate to work in excess of DCFH by the following reasons, (i) DCFH is expected to react with not only H_2_O_2_ but with all ROS generated after the stress-generating process, and (ii) the addition of the DCFH is done to samples with a high bacterial concentration 10^6^ CFU mL^−1^ and not for a single cell. Measurements of DCFH in bacterial suspensions will be done with the following protocol, which was also validated for flow cytometry, as explained below.

### DCFH - E. coli incubation

According to the literature, cells incubation time at 37 °C in presence of DCFH-DA should range between 30 and 60 minutes, to permit the diffusion of probes inside cells and the hydrolysis by endogenous esterase^38^,^39^. Nevertheless, these incubation times for samples containing solar irradiated *E. coli* may lead to modifications of physiological state of the cells, including ROS levels, due to metabolic activities during the incubation period. Therefore, a large incubation period could lead to false-positive or higher signal detection by an increment of ROS concentration that doesn’t represent the real state of the cells under evaluation. For this reason, this work is proposing the use of hydrolyzed DCFH-DA to DCFH as a tool that will also permit decreasing incubation times with bacteria, as esterase internal step is skipped.

**Figure 5:**
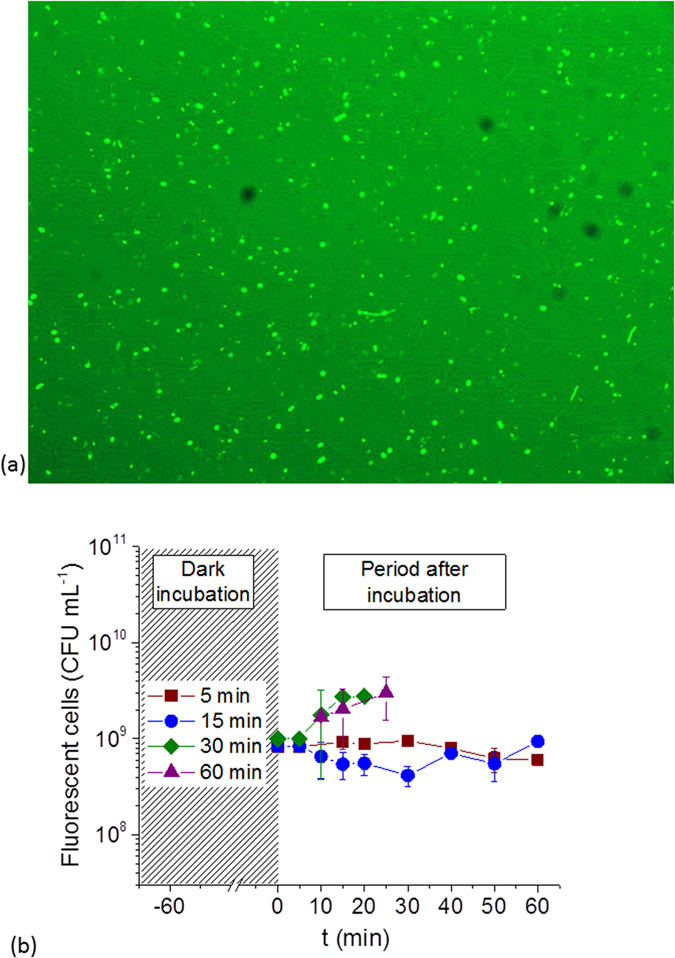
Incubation time of *E. coli* cells loaded with DCFH. (**a**) 40x fluorescence microscope photograph of 10^9^ CFU mL^−1^ *E. coli* sample loaded with 10 μM DCFH an incubated 15 minutes at 37 °C in dark; (**b**) Fluorescent *E. coli* cells counts with 10 μM DCFH an incubated for 5, 15, 30, and 60 min over a post incubation period (0–60 min).

With the aim of determine if shorter periods are adequate for incubation of DCFH and *E. coli*, preliminary experiments were done using unaltered *E. coli* suspensions and a fluorescence microscope, for direct counting of number of fluorescent bacteria. This will also give an idea of the baseline fluorescence signal of non-altered bacteria and its stability over time. The incubation experiments were done as follows. 10 μM of DCFH and 10^9^ CFU mL^−1^ *E. coli* were incubated at 37 °C for different times, 5, 15, 30, and 60 min, in dark. The fluorescent bacteria were directly observed and numbered using a counting chamber of 25 μL of samples. After each observation the total amount of fluorescent bacteria per sample was determined. Figure 5(a) shows a photograph of the fluorescence cells after 15 min incubation. Cell counting was done immediately after the tested incubation times, additionally each sample was re-numbered at different post-incubation times to confirm the stability of the measurement (Fig. 5). The first count result (time 0 in Fig. 5(b)) for all incubation times (5, 15, 30, and 60 min) revealed the same concentration of fluorescent bacteria, 10^9^ CFU mL^−1^, which was the expected initial concentration of alive bacteria in the suspension. These results confirm that diffusion of DCFH inside bacteria and further reaction with intracellular ROS is very fast, for 5 and 15 min incubation, and it doesn’t change over time (up to 60 min incubation). Nevertheless, longer incubation periods, 30 and 60 min, generated an increment in the number of counted florescent cells over post incubation time. This confirms the hypothesis that longer incubation times could lead to modifications in cells states and in intracellular ROS levels. Therefore, an incubation time between 5 and 15 min was found ideal for this protocol, because it gives a stable measurement over time of unmodified ROS. We selected 15 min incubation, which avoids long incubation periods and guarantee stable fluorescent measurement by successful DCFH diffusion and ROS-reaction inside cells.

### Validation of DCFH protocol using flow cytometry (H_2_O_2_ as positive control)

**Figure 6:**
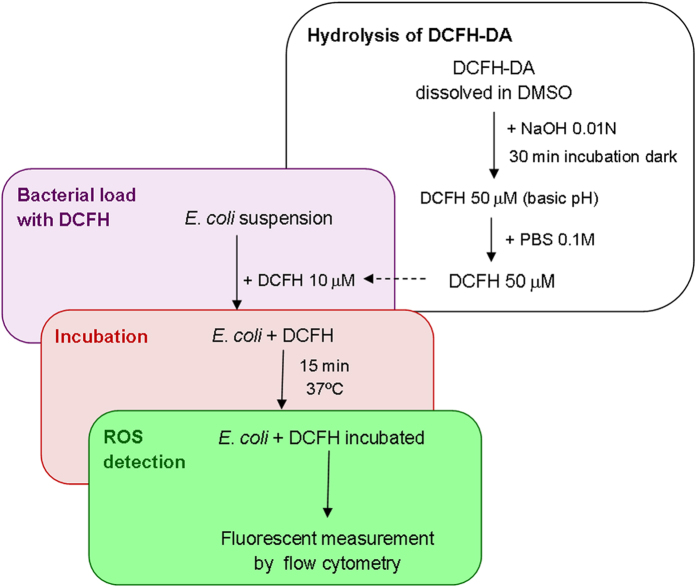
General scheme of proposed protocol for ROS detection in *E. coli* cells using DCFH-DA previously hydrolyzed as ROS-probe. Briefly, chemical hydrolysis of DCFH-DA to obtain DCFH, addition of DCFH to bacterial suspension, incubation period and fluorescence detection by flow cytometry.

**Figure 7:**
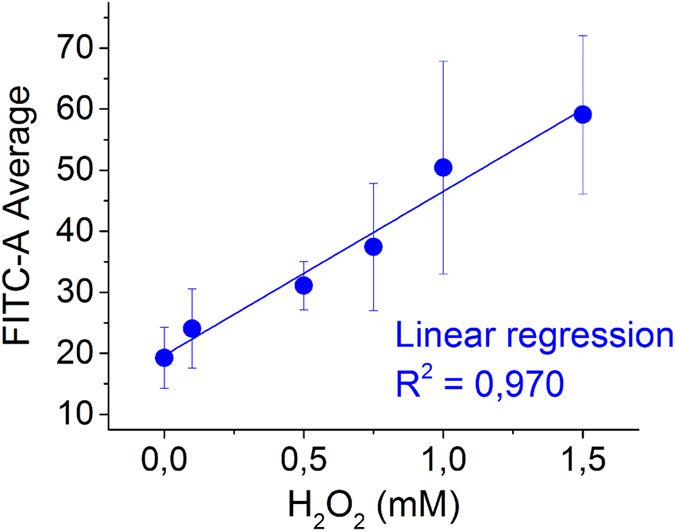
Validation of the internal ROS detection protocol using H_2_O_2_ as positive control. Fluorescent signal (excitation/emission: 488/530 nm) of *E. coli*-10 μM DCFH exposed to different concentrations of H_2_O_2_ by flow cytometry.

The proposed modified protocol is summarized in Fig. 6. Its validation was done by measuring the fluorescence intensity of bacteria exposed to oxidative stress of added H_2_O_2_ using a flow cytometer. Bacterial suspension cells (10^8^ CFU mL^−1^) were exposed for 10 minutes in dark to H_2_O_2_ (0, 0.1, 0.5, 0.75, 1, and 1.5 mM). Then samples were taken and loaded with 10 μM DCFH, incubated according to the above protocol and measured in the flow cytometer. Figure 7 shows the FITC-A average value of 50 000 cells reaching detector. The fluorescence intensity increased linearly with added H_2_O_2_. This result shows that the whole protocol works without any limitation in the flow cytometer, as well as H_2_O_2_ diffusion into cells was correctly happening. The calibration curve has a good linear response in the range of tested concentrations. In addition, the high resolution of the equipment is clear, as FITC-A values were lower than 100.

### Bacterial intracellular ROS formation during solar exposure

**Figure 8:**
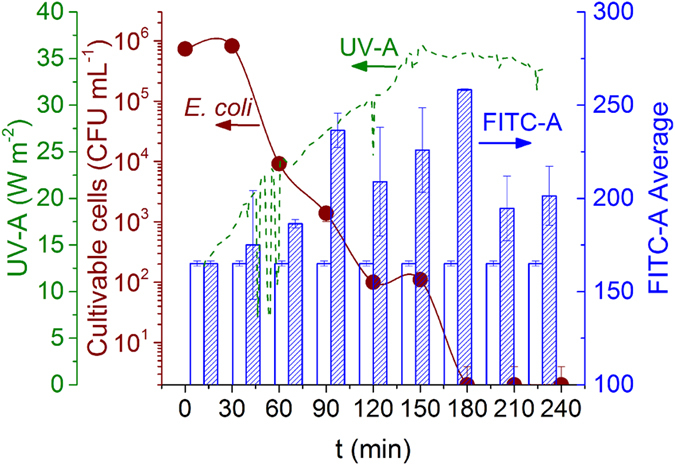
Solar water disinfection of *E. coli* within distilled water. Inactivation curve of *E. coli* (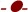), UV-A irradiance (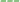) during solar irradiation, normalized FITC-A (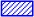), reference FITC-A value for control samples (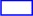).

During solar water disinfection experiments (four replicates), samples were taken every 30 min over 4 hours. Each sample was evaluated for viable *E. coli* counts and ROS determination using the proposed DCFH protocol (Fig. 8). *E. coli* concentration as a function of solar exposure time showed the typical SODIS inactivation curve, where a small shoulder for the first 30 min followed by a close to linear decay until reaching the detection limit (not counted colonies) at 180 min. Water temperature varied from 20 to 27 °C along the experiment; solar UV-A irradiance increased from 12.7 W m^−2^ (t = 0 min) to 36.1 W m^−2^ (t = 155 min), and then remained almost constant to the end of experiment.

For ROS-determination, the protocol shown in Fig. 6 was used. Each flow cytometry run was done against one control sample containing bacteria not exposed to solar radiation and treated with the same DCFH-DA protocol. This was used as a reference of ROS baseline against solar induced ROS measurements in irradiated samples. Baseline fluctuation of control samples’ FITC-A was used as a reference to normalize the signal of irradiated cells; results of normalized FITC-A are shown in Fig. 8. A significant increase in FITC-A for samples exposed to more than 60 min of solar radiation was observed; after 60 min the higher level of FITC-A remained nearly constant until the end of the experiment. According to these results, we can conclude that a solar induced generation of ROS is produced after a certain time period of solar exposure. The addition of the probe after the irradiation makes it to react with stable ROS mainly, so that the signal attributed to less stable ROS could be missing in this protocol. Therefore, the increment on fluorescence signal could be attributed to the most stable ROS, like H_2_O_2_, formed during solar exposure at intracellular level, which was detected using the new DCFH protocol proposed in this work.

## Discussion

To our knowledge there are not previous studies on the measurement or detection of intracellular ROS generated inside solar irradiated bacteria. Molecular techniques for investigating intracellular mechanisms of SODIS have been explored mostly from the genetic point of view. These studies demonstrated that DNA damages can modify ROS and SOS responses in bacteria. Some contributions use mutagenicity and genetic approaches to identify near-UV (NUV, 320–400 nm) radiation effects on bacteria cells. Webb and Brown demonstrated that irradiation with either broad-spectrum NUV or monochromatic wavelengths in the NUV can cause specific damages to DNA in *E. coli* K12 AB2480^40^. Eisenstark studied the DNA mutations due to NUV in *E. coli*. They assessed an experimental correlation between intracellular ROS and NUV; i.e. hydrogen peroxide resulted to be generated by the irradiation of tryptophan with UV-A, thus providing at least one pathway for generation of ROS by UV-A^36^; specific UV-mutations observed were responsible for the increase in the flux of HO^•^ radicals^41^ and O_2_^•−^ action^42^. Moreover, NUV radiation was proven to block SOS responses to DNA damages in *E. coli* that could result in cells inactivation^43^.

The generation of intracellular ROS is promoted by the absorption of light of endogenous chromophores. In this sense, experiments with mutants *E. coli katF* gene (hydroperoxidase II) suggested that porphyrin components of the respiratory chain may act as endogenous photosensitisers^44^. Also, it was found that cytochrome overproducing strains (cloned *cydA* and *cydB* genes) were sensitive to broad-band UV-A radiation^45^. Berney *et al*. investigated the effect of artificial sunlight on *E. coli* using flow cytometry and viability stains to monitor six cellular functions: efflux pump activity, membrane potential, membrane integrity, glucose uptake activity, total ATP concentration and culturability. They observed at different solar flux values that efflux pump activity and ATP synthesis decreased significantly, the loss of membrane potential, glucose uptake activity and culturability, and the cytoplasmic membrane of bacterial cells became permeable^46^.

Oxygen has been also investigated since early as one of the key factors affecting SODIS process, since it is the inducer of ROS generation and oxidative stress in aerobic bacteria under UV-A or sunlight^47^. Oxygen has been identified as a requirement for efficient inactivation of faecal bacteria under sunlight showing that efficiency of *Enterococcus faecalis* inactivation was faster in air-equilibrated water than in anaerobic conditions^8^; even more, the extent of inactivation during illumination was directly related to the dissolved oxygen content of the water^48^. Beyond this, Mani *et al*. investigated *E. coli* disinfection by SODIS process using plate count method under aerobic conditions and ROS-neutralized ambient with sodium pyruvate supplemented medium. Cultivable population from ROS-neutralized conditions was slightly higher than those in air^49^. This result indicated that a fraction of the cells become sub-lethally injured during sunlight exposure to the extent that they were unable to grow aerobically.

As mentioned before, genetic studies strongly suggested the photo-generation of ROS in bacteria exposed to NUV, and therefore to sunlight. According to Imlay^50^, ROS are continuously formed in *E. coli* through the adventitious autoxidation of its redox enzymes; their accumulation is controlled by superoxide dismutases, peroxidases and catalases. Under not stressed environment, the balance of these ROS is in equilibrium so bacteria are viable and stable. Superoxide dismutase avoids the accumulation of O_2_^•−^ in bacteria converting it to H_2_O_2_ that is then removed by catalase. UV-A radiation has a detrimental effect to these enzymes^51^, thus permitting higher accumulation of O_2_^•−^52. Microorganisms are vulnerable to elevated levels of intracellular ROS and it is considered one of the main pathways of bacterial inactivation during solar exposure.

The results of this work show for the first time an experimental evidence of the accumulation of intracellular stable ROS in *E. coli* during solar irradiation. This is a clear proof that oxidative stress was increased during the action of natural solar radiation. Previous reported studies on the bactericidal mechanism of SODIS showed indirect evidence that ROS were the main cause of bacterial inactivation, while the present contribution reports on new evidence of ROS generation over the basal levels (Fig. 8).

For the first time, this modified protocol for intracellular ROS detection was proposed and validated for *E. coli*. The use of hydrolyzed DCFH-DA and a reduction of incubation time permitted detection of intracellular ROS in solar irradiated *E. coli*. This protocol can be used for other bacteria, to investigate the influence of other stress factors, with the advantage of having a fast response and a high capability of detecting low levels of ROS, due to the fast reactivity of the hydrolyzed probe and the high sensitivity of flow cytometry, respectively.

## Additional Information

**How to cite this article**: Castro-Alférez, M. *et al*. Intracellular mechanisms of solar water disinfection. *Sci. Rep.* **6**, 38145; doi: 10.1038/srep38145 (2016).

**Publisher's note:** Springer Nature remains neutral with regard to jurisdictional claims in published maps and institutional affiliations.

## Figures and Tables

**Figure 1:**
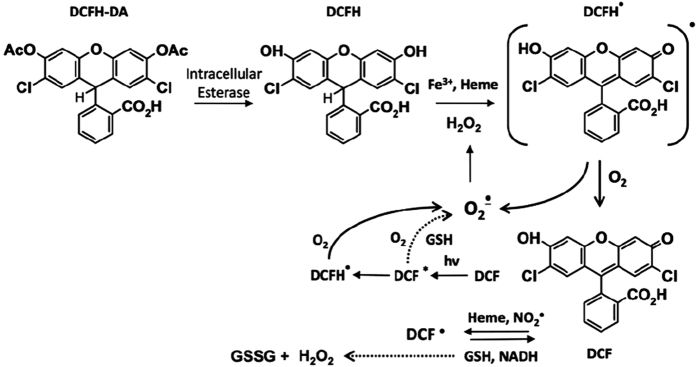
Formation of fluorescent product DCF. Intracellular reactions from DCFH-DA and redox cycling involving ROS and others intermediates to generate the final product, DCF. Imaged reproduced from ref. 24 Copyright 2014, Mary Ann Liebert, Inc.
